# Differential contributions of approximate number system, number line estimation, and working memory to mathematical skills in preschool and primary school

**DOI:** 10.1007/s00426-025-02144-x

**Published:** 2025-09-11

**Authors:** Vroni Hischa, Korbinian Moeller, Katja Seitz-Stein, Frank Niklas

**Affiliations:** 1 Department of Psychology, Catholic University of Eichstaett-Ingolstadt, Eichstaett, Germany; 2 Department of Mathematics Education, Loughborough University, Louhborough, UK; 3 LEAD Graduate School & Research Network, University of Tuebingen, Tuebingen, Germany; 4 Department of Psychology, University of Munich (LMU), Munich, Germany

## Abstract

Early mathematical skills predict later mathematical skills and general school achievement. The approximate number system (ANS), number line estimation, and working memory (WM) were found to be important predictors of the development of mathematical skills. However, studies specifying contributions of ANS, number line estimation, and WM at developmental levels are scarce. Therefore, the current study aimed to evaluate differential contributions of ANS, number line estimation, and WM to mathematical skills in preschool and primary school children. German preschool (*N* = 68, *M* = 6 years) and primary school children (*N* = 66, *M* = 9 years 1 month) completed an age-appropriate test for mathematical skills, a non-symbolic comparison task, a number line estimation task, WM span tasks, and a nonverbal intelligence task. Results indicated that ANS accuracy, number line estimation performance, and WM capacity were significant predictors for mathematical skills in both groups when controlled for influences of sex and nonverbal intelligence. Importantly, we also observed that only number line estimation performance contributed differentially to mathematical skills as it was a significantly stronger predictor in primary school children as compared to preschool children. In sum, these results suggest that the start of formal instruction on symbolic numerical representations in primary school is reflected in the contribution of related task performance to overall mathematics. To extend current findings, future studies may focus on differentiating age groups in a more fine-grained way to evaluate developmental trajectories of specific contributions of cognitive variables.

## Introduction

Several domain-specific numerical and domain-general cognitive predictors contribute to the development of mathematical skills (see Ashkenazi & Adi, 2023; Martin et al., 2014; Passolunghi & Lanfranchi, 2012; Purpura et al., 2017). Domain-specific numerical predictors include the approximate number system (ANS) and number line estimation performance (e.g., von Aster & Shalev, 2007). The ANS is an innate, nonverbal system that enables imprecise representations of quantities and thus their comparison (Halberda & Odic, 2015; Libertus, 2015; Mussolin et al., 2012; Starr & Brannon, 2015). The number line estimation task requires participants to indicate the position of a given target number on an unscaled number line. Performance on this task was repeatedly observed to predict current as well as future mathematical skills (SchnEider et al., 2018). In contrast to these domain-specific numerical predictors, working memory (WM) is a domain-general predictor of mathematical skills (Peng et al., 2016; Raghubar et al., 2010).

Importantly, however, contributions of ANS, number line estimation, and WM to the development of mathematical skills have hardly been investigated jointly, especially regarding potential differences in their contributions to mathematical skills at different levels of mathematical development (see Gimbert et al., 2019). Accordingly, the aim of this study was to evaluate such potential differential contributions of ANS and number line estimation as domain-specific predictors and WM as a domain-general predictor to the development of mathematical skills in preschool and primary school children. In the following, we will first review previous results on (differential) contributions of ANS, number line estimation, and WM to mathematical skills, before we derive our research questions and how we pursued them in more detail.

### ANS and mathematical skills

The ANS allows the approximate representation and processing of non-symbolic numerical quantities (Halberda & Odic, 2015; Starr & Brannon, 2015). Accordingly, ANS accuracy is often measured using non-symbolic comparison tasks (Dietrich et al., 2015). In these tasks, participants have to indicate, for example, which one of two presented dot arrays contains more dots without counting. This comparison is ratio-dependent and follows the Weber law (Dehaene, 1997, 2003; Dietrich et al., 2015; Mundy & Gilmore, 2009; Whalen et al., 1999).

Numerous studies reported an association between ANS accuracy and mathematical skills in preschool and primary school. Additionally, ANS accuracy was also identified as a longitudinal predictor for mathematical skills (Bonny & Lourenco, 2013; Libertus et al., 2011, 2013a; Malone et al., 2020; Van Marle et al., 2014). Therefore, the ANS is discussed to serve as a basis for the development of later mathematical skills (Chu et al., 2015; Feigenson et al., 2013; Halberda et al., 2008; Piazza, 2010). In particular, a more accurate ANS, which means a more precise representation of non-symbolic quantities, may facilitate associations of non-symbolic quantities with their corresponding symbolic numbers and therefore underlie later symbolic mathematical skills. However, this special role of ANS is debated (Chen & Li, 2014; De Smedt et al., 2013; Fazio et al., 2014; Feigenson et al., 2013; SchnEider et al., 2017).

Moreover, it needs to be noted that some ANS tasks draw on more general cognitive abilities such as intelligence or WM (Dietrich et al., 2015), for instance, when dot arrays are presented successively, mixed up or with their cumulated area incongruent to the total amount of dots. However, even when controlling for such general cognitive abilities, studies reported significant associations of ANS accuracy with mathematical skills in preschool and primary school children (Chen & Li, 2014; Feigenson et al., 2013), whereas others do not (Caviola et al., 2020; Coolen et al., 2022; Libertus et al., 2013b).

Accuracy of the ANS improves as children grow older (Halberda & Feigenson, 2008; Halberda et al., 2012). For example, quantities with a ratio of 3:2 can be distinguished by 3-year olds, but only six-year olds will successfully differentiate between quantities with a ratio of 7:6 (Halberda & Feigenson, 2008). Related to this, it is unclear whether and to what extent the role of ANS changes with the onset of formal mathematics instruction in primary school (Inglis et al., 2011). In some studies, significant relationships between ANS and mathematical skills were only found for preschool children, but not for school aged children (Cai et al., 2018; Gimbert et al., 2019; Xenidou-Dervou et al., 2017). In contrast, other studies report a persisting significant relationship between ANS and mathematical skills even after preschool (Fazio et al., 2014; Halberda et al., 2012; Lyons & Beilock, 2011).

### Number line estimation and mathematical skills

In the number line estimation task, a target number has to be located on an unscaled number line with only the start and endpoint given (Morris et al., 2022; Nazari et al., 2021). Many studies conducted with preschool and primary school children reported significant concurrent as well as longitudinal associations between number line estimation performance and other mathematical skills such as counting or written arithmetic. In particular, better number line estimation performance was associated with better development of more advanced mathematical skills (SchnEider et al., 2018). Accordingly, number line estimation performance is considered a significant predictor of the development of more complex mathematical skills (Geary, 2011; Gimbert et al., 2019; SchnEider et al., 2018; Siegler & Lortie‐Forgues, 2014). Similar to the case of the ANS, Ünal et al. (2024) point out that general cognitive abilities, such as intelligence and WM, should be controlled for when investigating contributions of number line estimation to mathematical skills, to prevent an overestimation.

The number line estimation task requires participants to associate symbolic with non-symbolic magnitudes, as a symbolic target number has to be positioned on an unscaled number line (for which only the start and end point are given, e.g., 0 and 100). In line with this, some studies reported that number line estimation might mediate the contribution of ANS to mathematical skills. However, studies investigating this are scarce and provide inconsistent results (e.g., Fazio et al., 2014; Gimbert et al., 2019). Based on their meta-analysis, Schneider et al. (2018) suggested that the association between number line estimation and mathematical skills increases between the age of 4 to 14 years.

### WM and mathematical skills

WM is a cognitive ability for short-term storage and manipulation of information (Baddeley, 2003). Baddeley’s WM model (2003) differentiates three components: the central executive (CE) as control and manipulation component and two subcomponents, the phonological loop (PL) for processing auditory information and the visuo-spatial sketchpad (VSSP) for processing visual information. There is empirical evidence that these three components can be distinguished by the age of 4 years (see Alloway et al., 2006).

In general, better WM capacity is positively correlated with mathematical skills and was found to predict them longitudinally in preschool and primary school (Cragg & Gilmore, 2014; De Smedt et al., 2009; Peng et al., 2016; Raghubar et al., 2010). Interestingly, most studies focus on contributions of single components, mainly the central executive of WM. Moreover, there are first studies jointly considering WM, ANS and number line estimation and their contributions to mathematical skills (Gimbert et al., 2019; Toll & Van Luit, 2014). For instance, in a study by Gimbert et al. (2019), number line estimation performance was found to mediate the contribution of ANS to mathematical skills in 5-year-olds. In 7-year-olds, however, the CE took over this mediating role. Findings by Price and Wilkey (2017) and Bull et al. (2018) further support the assumption of CE as a mediator between ANS and mathematical skills in children.

### The current study

Accordingly, this study aimed at better understanding potential differential contributions of ANS, number line estimation, and WM to mathematical skills in preschool and primary school children. Several meta-analyses have established that ANS, number line estimation, and WM each play an important role for mathematical skills (e.g., Chen & Li, 2014; Peng et al., 2016; Ünal et al., 2024), but hardly any study considered (differential) contributions of these variables jointly. However, this seems highly desirable considering the model by von Aster and Shalev (2007) describing the development of numerical representation.

The model is hierarchically organized and suggests contributions of ANS, number line estimation, and WM to the development of mathematical skills. On a first level, the model posits that the ANS provides basic meaning to numerosities, which is a prerequisite to link a perceived magnitude to a corresponding symbolic representation. On the second level, children get to know number words as a form of symbolization before, on the third level children are introduced to Arabic digits, as another form of symbolization when they start formal mathematics instruction in primary school. Number words and digital-arabic symbolization are, once again, prerequisites for the development of a mental number line on the fourth level of development. von Aster and Shalev (2007) further assume that the development of these different numerical representations is influenced by general cognitive abilities, such as WM, as a necessary resource. Importantly, however, the model does not specify whether influences of ANS, number line estimation, and WM are uniform across age or whether there are selective contributions of these variables depending on the level of numerical development.

However, there are only few studies that investigate contributions of ANS, number line estimation, and WM to the development of mathematical skills across different age groups and when they did, results were heterogeneous (Cai et al., 2018; Gimbert et al., 2019). Hence, our study investigates potential differential contributions of these variables to mathematical skills by comparing children from preschool and primary school. Comparing children from these two age groups is of special interest as the start of primary school and therefore formal mathematics instruction falls in-between. There is no mandatory mathematics curriculum in Germany before the start of primary school. In particular, as processing of and operating on symbolic numerical information is only instructed in primary school, we assumed an impact of formal schooling and, therefore, expected a change in the contributions of ANS, number line estimation, and WM to children’s mathematical skills, as suggested by the model of von Aster and Shalev (2007).

In line with previous evidence, we expected ANS, number line estimation, and WM to significantly predict mathematical skills in preschool and primary school children – even when controlled for influences of general cognitive ability (Halberda et al., 2008; Libertus et al., 2013a; Malone et al., 2020; Van Marle et al., 2014). Moreover, with the start of formal mathematics instruction in primary school, children regularly and intensively deal with symbolic numbers, they expand their knowledge about magnitudes and get to know scaled number lines (Staatsinstitut für Schulqualität & Bildungsforschung München, 2023). Against this background, one might well expect that number line estimation performance should be a more important predictor of mathematics skills in primary school children compared to preschool children because symbolic representations are only introduced systematically in primary school (SchnEider et al., 2018; von Aster & Shalev, 2007). The opposite could be hypothesized for contributions of the ANS as the model of von Aster and Shalev (2007) postulates that the ANS should be involved more in numerical development in preschool as compared to primary school.

## Method

### Participants

68 preschoolers (36 females, *M* = 6 years, *SD* = 5 months, range: 4 years 5 months – 6 years 10 months) and 66 primary school students (35 females, *M* = 9 years 1 month, *SD* = 5 months; range: 8 years 5 months – 10 years 4 months) participated in this study. Children were recruited from seven preschools and one primary school in Germany. Institutions were informed about the study via e-mail and phone calls, after which parents were sent an information letter. Only children who were willing to participate and had written parental consent took part. Three preschoolers were excluded from the original sample due to insufficient German language skills or unwillingness to participate during the assessment. The ethics committee of the Catholic University of Eichstaett-Ingolstadt approved the study.

### Tasks and materials

#### Mathematical skills

In preschool, data collection took place between February and June. The subtests *sequence of numbers*, *numerical knowledge*, *number concept*, *number seriation*, *number comparison* and *quantity comparison* of the MBK 0 (Krajewski, 2018) were used to assess mathematical skills.

In primary school, data collection took place in February and March. Participants were in third grade and thus assessed with the DEMAT 2 + (Krajewski et al., 2020), a standardized test for the assessment of mathematical skills based on the curricula of the federal states in Germany with subtests, for example, on *number characteristics*, *addition*, or *subtraction*. In both groups, z-standardized raw scores were used for data analysis. Internal consistency was $\alpha = .75$ for the MBK 0 in preschool and $\alpha = .84$ for the DEMAT 2 + in primary school and therefore comparable to the consistencies reported by Krajewski (2018) and Krajewski et al. (2020).

#### Approximate number system

ANS accuracy was assessed by a non-symbolic comparison task using Panamath (Halberda et al., 2008) on a 14’’ laptop. The task was used for preschool and primary school with exactly the same task characteristics. In each trial, an array of yellow dots (left) and blue dots (right) were presented next to each other for 2000 ms. Then a pixel mask appeared for 200 ms. Children had to indicate which of the arrays had more dots by pressing a yellow or a blue key (“F” and “J” on a QWERTZ-keyboard) as soon as they knew the answer. Each array contained between 10 and 30 dots, so that the number of dots could not be counted within the time limit. The average dot diameter was 36 pixels with a maximum variation of 25%.

In half of the trials, the yellow dot array contained more dots, in the other half, the blue one did. As soon as the answer was given, the next trial started after an interstimulus interval of 1000 ms. Five ratios were used (3.0, 2.0, 1.5, 1.3, and 1.2) with 8 trials each (Elliott et al., 2019; Libertus et al., 2011, 2013a, 2013b; Van Herwegen et al., 2017). The examiner gave an oral instruction using three practice trials (ratio 3.0) including feedback on the correctness of the response. Afterwards, no more feedback was given, and Panamath started with four beginner trials (ratio 3.0) and then 40 test trials.

As suggested by Dietrich et al. (2015), we used trials without and with size control. In half of the trials, the dot arrays were not size-controlled, i.e., all dots had the same average dot size and hence the cumulated area of the dots of each array was proportional to their number (congruent trials). In the other half of the trials, the dot arrays were size-controlled, i.e., the average dot size in the array with more dots was smaller than the one in the array with less dots and the cumulated area of the dots in both arrays was equal (incongruent trials). These control mechanisms were used to make sure that it was ANS, and not visual skills, that were measured (Dietrich et al., 2015).

Z-standardized percentage of correct answers and Weber Fraction *w* over all trials were highly correlated in preschool ($r = -.98, p < .001$) and primary school ($r = -.94, p < .001$). Thus, all further analyses were carried out using the percentage of correct answers, henceforth referred to as ANS accuracy. Furthermore, a Wilcoxon signed-rank test indicated no significant differences in ANS accuracy between trials without and with size control (sc) in preschool (${Mdn}_{without sc} = 90.00; {Mdn}_{with sc} = 90.00; z = -0.91, p = .363, n = 68$) and primary school (${Mdn}_{without sc} = 95.00; {Mdn}_{with sc} = 95.00; z = -0.84, p = .400, n = 66$). Hence, ANS accuracy over all trials was used for further analyses. Internal consistency was $\alpha = .66$ in preschool and $\alpha = .51$ in primary school. However, low reliability is common for tasks assessing ANS in children (e.g., Dietrich et al., 2015; Krajcsi et al., 2024).

#### Number line estimation

Number line estimation performance was assessed using a number line estimation task. Most studies with this task use a paper–pencil format (Gimbert et al., 2019; Sasanguie et al., 2016) or a digital version on a computer screen where participants have to click with a mouse to indicate their answer (Fazio et al., 2014; Lee et al., 2022).

In this study, however, we used a custom tablet application implemented in PsychoPy (Peirce et al., 2019) for a more intuitive response format. On a 14’’ tablet, a black 25,5 cm line was shown on a white background. The left end was marked with a 0 underneath, and the right end was marked with either a 10 or 100 underneath. In each trial, a number was shown in the upper center of the screen. Children were asked to tap on the line where they thought the number should go (e.g., “If 0 is here and 10 is here, where should 5 go?”). No feedback was given. Preschoolers responded to three items (2, 4, and 7) on a number line from 0 to 10 and had to read the numbers out loud. In case the numbers were not identified correctly, the examiner read them out. Primary school students completed the same three items, but without reading them out loud. In addition, they responded to six items on a number line from 0 to 100 (i.e., 2, 4, 6, 18, 42, and 71, taken from Siegler and Opfer (2003)).

To make sure that the whole number line was considered, example trials (placing 5 on the number line from 0 to 10 and placing 50 on the number line from 0 to 100) were used during task instruction (e.g., “Down here you see a line. At the beginning of the line is 0. At the end of the line is 10.”) (see Morris et al., 2022). In preschool, the examiner explained the number line in every trial, like in the example trial.

Mean percentage of absolute error (PAE) over all trials was used for data analyses: $\text{PAE }= \left[\left|Estimate - Actual\; Number\right| / Scale\; of\; Estimates\right]\times 100$(Cai et al., 2018; Gimbert et al., 2019; Sasanguie et al., 2016). Internal consistency based on the PAE was $\alpha = .62$ for the three items in preschool and $\alpha = .67$ for the nine items in primary school.

#### Working memory

The WM components CE, PL and VSSP were assessed using the tablet-based application EI-MAG (Oesterlen et al., 2016) and hence in more detail than in other studies (Gimbert et al., 2019; Toll & Van Luit, 2014; Van de Weijer-Bergsma et al., 2015).

The subtests *word span backwards*, *digit span backwards*, *object span*, and *counting span* were used to measure the capacity of CE. In the *word span backwards*, an audio sequence of monosyllabic words was presented and afterwards, corresponding pictures had to be tapped in a picture matrix in reverse order of presentation. The *digit span backwards* worked analogously but used digits and a number pad instead. In the *object span*, sequences of objects were presented visually. For each of them, participants had to decide whether an animate or inanimate entity was shown (secondary task). After each sequence, all objects had to be recalled in order of presentation by tapping the corresponding pictures in a picture matrix (primary task). The *counting span* subtest was similar to the counting span subtest of the AGTB 5–12 (Hasselhorn et al., 2012). In this task, sequences of pictures were shown. In each picture, participants had to count the number of specific target objects and then enter the corresponding number using a number pad. Participants had to do this for each picture separately. At the end of a sequence, another number pad was shown and participants had to tap all the numbers counted before in order of presentation.

The capacity of the PL was assessed using the *word span* (monosyllabic and trisyllabic) and the *digit span*. These subtests worked the same way as the *word span backwards* and the *digit span backwards*, but forwards.

The *corsi block* and the *matrix* subtests were used to measure the capacity of VSSP. In the *corsi block* subtest blocks were shown in a randomly arranged order. Single blocks temporary turned orange one after another. The respective sequences of blocks turning orange had to be recalled by tapping the corresponding blocks in the order in which they had turned orange before. The *matrix* subtest was similar to the matrix subtest of the AGTB 5–12 (Hasselhorn et al., 2012). In this task, matrices with patterns of black cells were presented. After each of these matrices, a white matrix was shown and participants had to tap all the cells that had been black in the previous matrix.

At the beginning of each subtest, a practice phase was implemented to check whether the instruction had been understood correctly. If needed, the application prompted the examiner to provide additional verbal 1:1 instruction. In all subtests, span length increased in an adaptive manner. For preschoolers trisyllabic word span, object span, and counting span were omitted to take the general attention span and cognitive resources of this age group into account and to keep the assessment at an economic length. For each subtest, the mean length of the longest sequences that were recalled correctly was used as subtest span. For each WM component, the mean of the subtest spans for this component (i.e. CE, PL, VSSP) was calculated.

For WM in general, we then used the mean of the three components. For 10 preschoolers and 9 primary school students not all subtests per component were completed. In this case, the mean of the remaining subtests was used as WM measure for the respective component. If no subtest of a component was completed, the mean of the remaining component spans was used as WM span. Reliability for WM span was $\alpha = .76$ based on the six WM subtests in preschool (*word span backwards*, *digit span backwards*, *word span monosyllabic*, *digit span*, *corsi block*, *matrix*) and $\alpha = .74$ based on the nine WM subtests in primary school (*word span backwards*, *digit span backwards*, *object span*, *counting span*, *word span monosyllabic* and *trisyllabic*, *digit span*, *corsi block*, *matrix*). Overall, obtained internal consistencies were comparable to the ones reported by Oesterlen et al. (2018). Reliability for the CE score was $\alpha = .72$ based on the two CE subtests in preschool and $\alpha = .47$ based on the four CE subtests in primary school.

#### Control variables

Children’s age in months, sex, and nonverbal intelligence were considered as control variables (see Cai et al., 2018; Dietrich et al., 2015). For the assessment of nonverbal intelligence, the subtest nonverbal intelligence of BUEVA-III (Esser & Wyschkon, 2016) was used in preschool and the same subtest of BUEGA-II (Esser et al., 2021) was used in primary school. In the subtest nonverbal intelligence of BUEVA-III, four to five images were presented. Participants had to decide which of the images did not fit in with the others. In the subtest nonverbal intelligence of BUEGA-II, incomplete matrices were presented. Participants had to indicate which image out of several given alternatives fitted to complete the matrix.

For data analyses, z-standardized raw scores of these subtests were considered. Internal consistency was $\alpha = .72$ in preschool and $\alpha = .85$ in primary school and therefore comparable to the internal consistencies reported by Esser and Wyschkon (2016) and Esser et al. (2021).

### Procedure

Preschoolers took part in two one-on-one sessions (approx. 35 min) in a quiet room in their kindergarten. In the first session, they completed the subtests of MBK-0, three subtests of EI-MAG, and the number line estimation task. In the second session, they were assessed with the subtest nonverbal intelligence of BUEVA-III, another three subtests of EI-MAG, and the non-symbolic comparison task. Both sessions took place within one week between February and June except for three children who did the second session two months after the first session due to illness. We compared the results of these three children to the remaining sample of preschoolers applying the procedure suggested by Crawford and Garthwaite (2002) and Crawford and Howell (1998). Results indicated that performance of these three children on nonverbal intelligence, ANS accuracy, number line estimation performance, WM and CE capacity, and mathematical skills did not differ significantly from the rest of the sample. Thus, their data were included in the analyses.

Participants in primary school took part in a group session (approx. 90 min) and a one-to-one session (approx. 35 min) in a quiet room at their school. In the group session, they completed nine subtests of EI-MAG and the DEMAT 2 +. In the one-on-one session, they completed the subtest nonverbal intelligence of BUEGA-II, the non-symbolic comparison task, and the number line estimation task. Both sessions took place within one week.

### Statistical analyses

Statistical analyses were conducted using SPSS version 29.0.0.0 (IBM Corp., 2022). Descriptive and correlation analyses will be presented first.

Subsequently, we employed a hierarchical regression analysis to evaluate the predictive power of ANS accuracy, number line estimation performance, and WM capacity for mathematical skills while controlling for sex and nonverbal intelligence. In a first step, to evaluate potential differences in the contributions of these predictors between preschool and primary school children, we considered a predictor reflecting the two groups (coded 0 for preschool, 1 for primary school) and the interaction terms of this group variable with ANS accuracy, number line estimation performance, and WM span respectively. ANS accuracy, number line estimation performance, and WM span were z-standardized within the groups prior to the analysis. For mathematical skills, we considered the percentage of the maximum achievable scores reached by participants in the respective mathematical test.

We conducted a sensitivity analysis using G*Power (Faul et al., 2009) with $\alpha$ = 0.05 and a power of 0.90 for the hierarchical regression analysis (Lakens, 2022). This led to ${f}^{2}$ of 0.16 for the whole sample, indicating that with the current sample and design we should be able to observe moderate to strong effects.

A lot of studies investigating the role of ANS, number line estimation, and WM for mathematical skills focus on CE only, instead of WM more generally (see Gimbert et al., 2019; Toll & Van Luit, 2014). Therefore, we additionally ran a hierarchical regression analysis with z-standardized CE span instead of z-standardized WM span.

In a second step, we further evaluated if number line estimation and CE span mediate the association of ANS with mathematical skills differentially in preschool and primary school. Moreover, we expected that number line estimation/CE mediates the relationship between ANS and mathematical skills in preschool/primary school. Accordingly, we ran simultaneous mediation models to evaluate whether number line estimation performance or CE capacity mediated the association between ANS and mathematical skills. We used the PROCESS macro (version 4.2) for SPSS (Hayes, 2022) with a 95% confidence interval based on 5000 bootstrap samples. In all models, children’s age, sex, and nonverbal intelligence score were considered as covariates. Gimbert et al. (2019) and Fazio et al. (2014) used comparable sample sizes to our study to investigate a similar mediation.

## Results

### Descriptive and correlation analyses

Table 1 shows the results of descriptive and correlation analyses for age, sex, nonverbal intelligence score, ANS accuracy, PAE in the number line estimation task, WM span, and mathematical skills. As most variables were not normally distributed in both groups, Spearman’s $r$ was used. Mathematical skills correlated significantly with all other variables in preschool and primary school, except for sex in preschool and age in primary school. Furthermore, the nonverbal intelligence score was significantly associated with ANS accuracy in preschool and primary school. ANS accuracy and PAE in the number line estimation task correlated significantly in preschool, but not in primary school. The nonverbal intelligence score and WM span were significantly associated in preschool and primary school. ANS accuracy and WM span also correlated significantly in preschool and primary school. PAE in the number line estimation task did not correlate significantly with the nonverbal intelligence score and WM span in preschool, but both correlations were significant in primary school. Therefore, correlation analyses indicated some differences between the two groups regarding the pattern of associations between the variables assessed.

**Table 1:** Descriptive statistics and correlation coefficients between age, sex, nonverbal intelligence score, ANS accuracy, PAE in the number line estimation task, WM span, and mathematical skills in preschool and primary school

	*M*	*SD*	1.	2.	3.	4.	5.	6.	7.
1. Age in months	72.44 / 109.42	5.89 / 5.43	—	–.13	–.12	–.18	.04	–.19	.09
*p*				.152	.176	.072	.368	.066	.239
2. Sex	0.53 / 0.53	0.50 / 0.50	–.05	—	.03	.10	.18	–.08	–.40
*p*			.331		.397	.209	.071	.269	< .001
3. Nonverbal intelligence score	24.00 / 21.03	3.04 / 5.03	.18	.08	—	.30	–.28	.36	.42
*p*			.066	.265		.007	.012	.002	< .001
4. ANS accuracy	87.46 / 94.62	8.67 / 5.10	.09	.12	.33	—	–.13	.38	.30
*p*			.231	.169	.003		.145	< .001	.007
5. PAE in number line estimation task	18.34 / 6.13	10.78 / 3.30	–.13	.18	–.09	–.23	—	–.35	–.57
*p*			.142	.075	.242	.032		.002	< .001
6. WM span	2.74 / 3.79	0.47 / 0.61	.11	.11	.31	.34	–.03	—	.46
*p*			.190	.180	.005	.002	.399		< .001
7. Mathematical skills	35.32 / 24.23	7.14 / 7.76	.31	–.12	.26	.33	–.37	.50	—
*p*			.005	.158	.015	.003	< .001	< .001	

*Notes.* For *M* and *SD*, values for preschool are written on the left and values for primary school are written on the right. *M* and *SD* reflect raw scores obtained for the respective tasks that preschoolers and primary school students completed. Correlation coefficients for preschool are written below the diagonal. Correlation coefficients for primary school are written above the diagonal. One-tailed bivariate nonparametric correlations (spearman-rho). Sex was coded 0 for male and 1 for female participants

A comparison of the correlation coefficients showed significant differences between preschool and primary school children concerning the association of sex and mathematical skills ($z = 1.72, p = .042$) and of PAE in the number line estimation task and WM span ($z = 1.91, p = .028$) (Eid et al., 2017). In primary school, boys had significantly higher mathematical skills than girls and students with more accurate number line estimations had significantly higher WM spans. All other correlation coefficients were not significantly different between preschool and primary school children (all $\text{ps }\ge .071$).

### Hierarchical regression analyses

A hierarchical regression analysis was conducted with the percentage of the maximum achievable scores reached by participants in the respective mathematical test (MBK 0 or DEMAT 2 +) as criterion variable, to identify significant predictors for mathematical skills and potential developmental differences concerning these in preschool and primary school (see Table 2). Sex and nonverbal intelligence score were entered as predictors in Step 1. Then in Step 2, ANS accuracy, PAE in the number line estimation task, WM span, a group variable (preschool vs. primary school), and the respective interaction terms of ANS accuracy, PAE in the number line estimation task, and WM span with the group variable were added.

**Table 2:** Hierarchical regression analysis with mathematical skills as criterion variable and sex, nonverbal intelligence score, ANS accuracy, PAE in the number line estimation task, WM span, group, and the respective interaction terms as predictor variables

Variable	*B*	95% CI for *B*		*SE B*	β	*p*	*R* ^*2*^	*ΔR* ^*2*^
		*LL*	*UL*					
Model Step 1							.19	
Constant	78.87	74.45	83.30	2.24		< .001		
Sex	–11.12	–17.21	–5.03	3.08	–.28	< .001		
Nonverbal intelligence score	7.31	4.25	10.37	1.55	.37	< .001		
Model Step 2						< .001	.55	.37
Constant	83.55	79.54	87.56	2.03		< .001		
Sex	–9.57	–14.26	–4.89	2.37	–.24	< .001		
Nonverbal intelligence score	2.88	0.38	5.37	1.26	.15	.024		
ANS accuracy	4.07	0.43	7.71	1.84	.21	.029		
PAE in number line estimation task	–4.05	–7.46	–0.64	1.72	–.21	.020		
WM span	5.45	2.02	8.87	1.73	.28	.002		
Group	–11.15	–15.64	–6.67	2.27	–.29	< .001		
Interaction: ANS accuracy*group	0.83	–4.22	5.87	2.55	.03	.746		
Interaction: PAE in number line estimation task * group	–5.89	–10.82	–0.95	2.49	–.21	.020		
Interaction: WM span * group	–2.96	–8.11	2.20	2.61	–.11	.259		

*Notes.* Standardized regression coefficients *β* and adjusted *R*^2^. CI, confidence interval; *LL*, lower limit; *UL*, upper limit. Sex variable was coded 0 for male and 1 for female participants. The group variable was coded 0 for preschool children and 1 for primary school children. ANS accuracy, PAE in the number line estimation task, and WM span were z-standardized within the groups for this analysis. To check for potential suppression effects, we reran the regression analysis considering only one interaction predictor at a time. Importantly, this did not change the results. The interaction predictor of number line estimation and group was the only significant influence identified. This rules out potential suppression effects

In Step 1, the regression analysis indicated that sex and nonverbal intelligence were significant predictors, accounting for approximately 19% of the variance in children’s mathematical skills $[{R}^{2}= .198, \text{adj}. \;{R}^{2}= .186, F\left(2, 131\right)=16.19, p < .001]$. Inspection of the beta weights suggested that mathematical skills were higher for boys and for children with higher nonverbal intelligence.

In Step 2, the regression analysis revealed that inclusion of further predictors increased explained variance significantly to approximately 55 %$[{R}^{2}=.583, \text{adj}. {R}^{2}=.553, F\left(9, 124\right)=19.27, p <.001 ]$. In addition to the prevailing significant influences of sex and nonverbal intelligence, there were significant influences of ANS, number line estimation, WM, and group. Importantly, the significant main effect of number line estimation was qualified by a significant interaction with group.

**Fig. 1:**
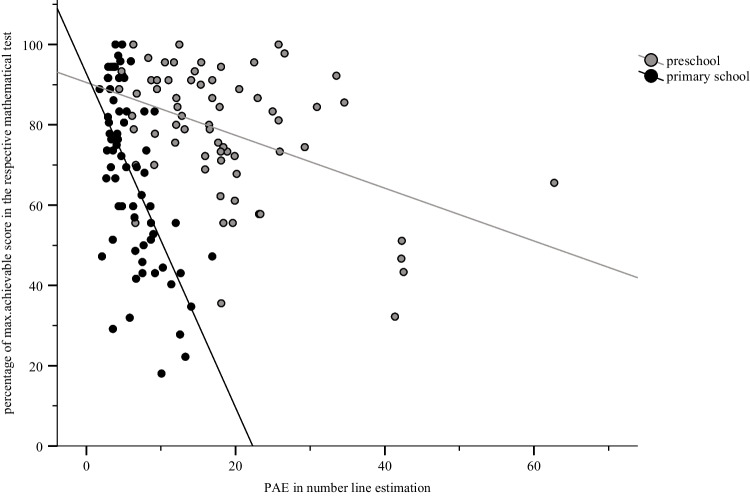
Associations between number line estimation and mathematical skills for preschool and primary school children. *Notes.* Each dot represents an individual participant. ${R}^{2}= .20$ for preschool children, ${R}^{2}= .41$ for primary school children

Closer inspection of the beta weights indicated that preschool children performed better than primary school children on the respective test for mathematical skills, meaning they achieved a higher percentage of the maximum achievable score in the respective test. Additionally, higher ANS accuracy and higher WM span predicted better mathematical skills irrespective of participant group. Importantly, however, the significant influence of the interaction predictor of group and number line estimation suggested that the influence of number line estimation on mathematical skills differed between groups. In fact, number line estimation performance was a significantly stronger predictor for mathematical skills for primary school children than preschool children (see Fig. 1).

Most of the cited literature concerning associations of WM and mathematical skills with ANS and number line estimation focuses on CE (see Gimbert et al., 2019; Toll & Van Luit, 2014). Thus, we also ran this hierarchical regression analysis with CE instead of WM (see Table 3).

**Table 3:** Hierarchical regression analysis with mathematical skills as criterion variable and sex, nonverbal intelligence score, ANS accuracy, PAE in the number line estimation task, CE span, group, and the respective interaction terms as predictor variables

Variable	*B*	95% CI for *B*		*SE B*	β	*p*	*R* ^*2*^	*ΔR* ^*2*^
		*LL*	*UL*					
Model Step 1							.19	
Constant	78.87	74.33	83.42	2.30		< .001		
Sex	–11.12	–17.38	–4.85	3.16	–.28	< .001		
Nonverbal intelligence score	7.31	4.16	10.46	1.59	.37	< .001		
Model Step 2						< .001	.54	.35
Constant	83.71	79.53	87.89	2.11		< .001		
Sex	–9.87	–14.75	–4.99	2.47	–.25	< .001		
Nonverbal intelligence score	3.39	0.83	5.95	1.29	.17	.010		
ANS accuracy	4.89	1.17	8.61	1.88	.25	.010		
PAE in number line estimation task	–3.71	–7.27	–0.16	1.79	–.19	.041		
CE span	3.82	0.28	7.36	1.79	.19	.035		
Group	–11.15	–15.84	–6.47	2.36	–.29	< .001		
Interaction: ANS accuracy*group	–0.004	–5.08	5.07	2.56	–.0002	.99		
Interaction: PAE in number line estimation task * group	–6.38	–11.36	–1.40	2.52	–.23	.012		
Interaction: CE span * group	–0.81	–5.91	4.29	2.58	–.03	.754		

*Notes.* Standardized regression coefficients *β* and adjusted *R*^2^. CI, confidence interval; *LL*, lower limit; *UL*, upper limit. Sex variable was coded 0 for male and 1 for female participants. The group variable was coded 0 for preschool children and 1 for primary school children. ANS accuracy, PAE in the number line estimation task, and CE span were z-standardized within the groups for this analysis. To check for potential suppression effects, we reran the regression analysis considering only one interaction predictor at a time. Importantly, this did not change the results. The interaction predictor of number line estimation and group was the only significant influence identified. This rules out potential suppression effects

In Step 1, results were virtually identical to the ones returned from the hierarchical regression on WM. The regression analysis indicated that sex and nonverbal intelligence were significant predictors accounting for approximately 19% of variance in children’s mathematical skills $[{R}^{2}= .198, \text{adj}. \;{R}^{2}= .185, F\left(2, 124\right)=15.32, p < .001]$. Again, inspection of the beta weights suggested that mathematical skills were higher for boys and for children with higher nonverbal intelligence.

In Step 2, the regression analysis revealed that inclusion of further predictors increased explained variance significantly to approximately 54% $[{R}^{2}=.572, \text{adj}.\; {R}^{2}=.539, F\left(9, 117\right)=17.40, p <.001 ]$. In addition to the prevailing significant influences of sex and nonverbal intelligence, there were significant influences of ANS, number line estimation, CE, and group. Again, the significant main effect of number line estimation was qualified by a significant interaction with group. Inspection of beta weights indicated identical predictions to the ones described for WM above.

### Mediation analyses

**Fig. 2:**
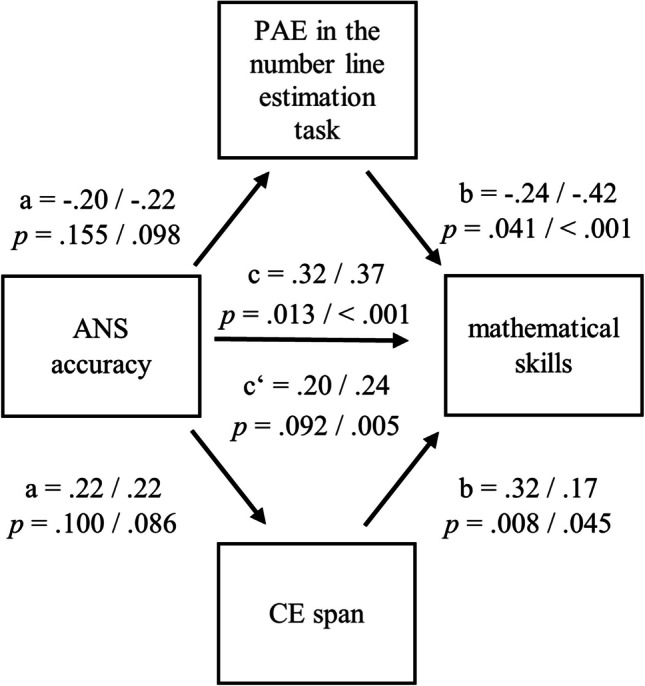
Mediation models for mathematical skills with PAE in the number line estimation task and CE span as mediators in preschool and primary school**.** *Notes*. Simultaneous mediation models with standardized coefficients and controlled for age, sex, and nonverbal intelligence score. Values for preschool are written on the left, values for primary school on the right. Z-standardized scores for mathematical skills were used

Mediation analyses run separately for preschool and primary school children indicated no significant effects of ANS accuracy on number line estimation performance and CE span (see Fig. 2). In contrast, the effects of number line estimation performance on mathematical skills and of CE span on mathematical skills were significant. The direct effects of ANS accuracy on mathematical skills were also significant. The indirect effect of ANS accuracy on mathematical skills was not significant in preschool children but was significant in primary school children. The confidence intervals for the standardized, total indirect effect ([−0.01, 0.26] for preschool; [−0.05, 0.29] for primary school), the specific indirect effect of PAE in the number line estimation task ([−0.01, 0.15] for preschool; [−0.06, 0.23] for primary school) and of CE span ([−0.02, 0.16] for preschool; [−0.01, 0.10] for primary school) indicated that the relationship between ANS accuracy and mathematical skills in preschool and primary school was neither mediated by number line estimation performance nor by CE span.

## Discussion

This study set off to evaluate the contributions of ANS, number line estimation, and WM to the development of mathematical skills with a specific focus on potential differential contributions associated with the start of formal schooling. In the following, we first summarize and discuss the findings on the contributions of ANS, number line estimation, and WM to mathematical skills before we elaborate on the observed differential contributions of number line estimation to mathematical skills in preschool and primary school. Afterwards, we discuss the results of the mediation of the association between ANS and mathematical skills by number line estimation and CE.

### Contributions of ANS, number line estimation, and WM to mathematical skills

The results of the current study indicated a significant contribution of ANS to mathematical skills. In both groups, higher ANS accuracy was a significant predictor of better mathematical skills. This is in line with previous evidence (Bonny & Lourenco, 2013; Fazio et al., 2014; Libertus et al., 2011; Malone et al., 2020; Van Marle et al., 2014). Higher ANS accuracy means more precise representations of non-symbolic quantities which are a crucial building block for the development of symbolic number representations. In this way, ANS may play a foundational role within the development of mathematical skills (Chu et al., 2015; Feigenson et al., 2013; Halberda et al., 2008; Piazza, 2010).

However, this role of ANS is debated in the literature (Chen & Li, 2014; Feigenson et al., 2013; SchnEider et al., 2017; Ünal et al., 2024). Methodological differences might explain heterogeneous findings, as typical ANS tasks also put demands on more general cognitive abilities (De Smedt et al., 2013; Dietrich et al., 2015; Fazio et al., 2014). However, it is important to note that Dietrich et al. (2015) suggested that paired non-symbolic comparison tasks (such as the one we used in this study) place fewer additional demands on WM than, for example, sequential non-symbolic comparison tasks. Moreover, in our study, ANS accuracy was still a significant predictor for mathematical skills in preschool and primary school children when controlling for nonverbal intelligence and WM or CE. Therefore, the ANS seems to have predictive power over and above these controlled variables in both groups. This is consistent with the findings by Chen and Li (2014) and with Fazio et al. (2014).

Our results also corroborate previous findings on the contribution of number line estimation to mathematical skills. In both groups, number line estimation performance was a significant predictor of mathematical skills (Geary, 2011; Gimbert et al., 2019; Sasanguie et al., 2012; SchnEider et al., 2018; Siegler & Lortie‐Forgues, 2014; Ünal et al., 2024). As such, more accurate number line estimations seem to predict better mathematical skills more generally and may thus serve as a foundation for the development of more advanced mathematical skills (SchnEider et al., 2018). In particular, performing the number line estimation task usually involves the application of other mathematical skills (see SchnEider et al., 2018 for an overview). Typically, estimating the position of a target number involves the use of reference points. In the beginning, children estimate the magnitude of a target number relative to the start point of the number line. Later, they will also use the endpoint and the midpoint as additional reference points allowing them to use proportion judgement strategies to solve the number line estimation task applying their relational knowledge about numbers (see Dackermann et al., 2015 for an overview; Slusser & Barth, 2017; Slusser et al., 2013). This may involve halving of the number range to establish the midpoint as a reference point (e.g., 50 in case of the 0 to 100 range). In a next step, children may establish whether the target number is larger or smaller than this reference point using magnitude comparison, before they need to decide how far to the left or right of the midpoint the target number is located, for instance by subtracting or adding the respective magnitude (see Link et al., 2014). In sum, this suggests that the contribution of number line estimation performance to other mathematical skills is driven by the fact that children actually apply different mathematical skills when solving the number line estimation task.

Finally, in both preschool and primary school, higher WM and CE capacity was a significant predictor of better mathematical skills (see also Cragg & Gilmore, 2014; Peng et al., 2016; Raghubar et al., 2010). This contradicts recent results by Gimbert et al. (2019) who identified WM/CE as a predictor for mathematical skills in primary school, but not in preschool. These divergent findings may be due to differences in the assessments of WM capacity. While Gimbert et al. (2019) used a single complex span task, we used several simple and complex span tasks. Therefore, WM components and in particular CE were assessed in more detail in the current study.

#### Differential contributions of number line estimation in preschool and primary school

A comparison of correlation coefficients indicated that the associations between control variables, ANS, number line estimation, WM, and mathematical skills did not differ between preschool and primary school children, with two exceptions. In primary school but not in preschool children, boys had significantly better mathematical skills than girls, which aligns with previous german studies (Niklas & SchnEider, 2012). Moreover, better number line estimation performance was associated with higher WM capacity in primary school compared to preschool.

Hierarchical regression analysis controlled for sex and nonverbal intelligence revealed significant differences between preschool and primary school for contributions of number line estimation performance to mathematical skills. Number line estimation performance was a significantly more important predictor for mathematical skills in primary school than in preschool. This finding is in line with our expectations and the meta-analysis by Schneider et al. (2018) which suggested that associations between number line estimation and mathematical skills increase from ages 4 to 14. With the beginning of formal mathematics instruction in primary school, children process and operate more on symbolic numerical representations, which in turn may improve their number line estimation performance. As number line estimation performance improves, for instance, the processing of numerical magnitudes but also the application of proportion judgement strategies (i.e., using the midpoint as a reference) become more precise and prevalent and might therefore facilitate the development of more advanced mathematical skills (SchnEider et al., 2018).

#### Neither number line estimation nor CE mediated between ANS and mathematical skills

In the current study, neither number line estimation performance nor CE mediated the relationship between ANS and mathematical skills in preschool and primary school. These findings are partly inconsistent to the ones by Gimbert et al. (2019). In their study, number line estimation performance was a partial mediator of the relationship between ANS and mathematical skills in preschool, whereas in primary school CE took over this role and became a full mediator. Furthermore, in the studies by Price and Wilkey (2017) and Bull et al. (2018), CE fully mediated the relationship between ANS and mathematical skills.

Again, methodological differences between studies might be a reason for these divergent findings. In addition to the differences in the CE tasks, Gimbert et al. (2019) focused on arithmetic and word problems, whereas our study considers a broader range of mathematical skills, including, for example, division and geometry. Contrary to the current study, Price and Wilkey (2017) and Bull et al. (2018) investigate older age groups or larger age spans respectively (12 years and 5 to 12 years).

#### Limitations

There are some limitations to be considered when interpreting the results of our study. First, it needs to be mentioned that the sample assessed in the current study is relatively small. The initial sample size was notably reduced due to sickness of participants, missing parental consent or insufficient German language skills. Sensitivity analysis showed that medium sized effects can be detected in the regression analyses with a power of 0.90, meaning that all effects observed should be about this size or larger. Furthermore, based on the results of our cross-sectional study no causal statements about developmental trajectories are admissible.

Additionally, it is worth noting that there were indications of a ceiling effect for ANS accuracy in both the preschool and primary school sample. This means that based on the ratios of the pairs of dot arrays and the presentation time used in our study, participants were well able to decide which array contained more dots indicating that the used non-symbolic comparison task might have been very easy. Previous, studies used comparable ratios but with shorter stimulus presentation times than our study (e.g., Fazio et al., 2014). Hence, in future studies, it would be desirable to include a broader range of ratios and/or shorter stimulus presentation times should be employed. Due to these ceiling effects, the effects of ANS may be underestimated.

Due to differences in educational systems, formal mathematics instruction starts at different ages for children in different countries (e.g., Niklas et al., 2018). This study was conducted in Germany, where there is no mandatory mathematics curriculum before children start primary school at about the age of six or seven years. This is different to other countries such as France, the UK or the US. In essence, this means that daycare facilities for children prior to primary school can decide on their own if and if so, what mathematics instruction they provide. In most of the cases, this implies that children in Germany only receive systematic instruction on processing of and operating on symbolic numerical information when they start primary school.

An earlier start of formal mathematics instruction based on a standardized curriculum may facilitate early mathematical learning and may, for example, strengthen the association between non-symbolic and symbolic representations earlier. Therefore, the transferability of the results to samples from other countries with differing educational systems needs to be investigated in future studies. Furthermore, based on the design of the current study, it is important to note that general influences of developmental maturation cannot be ruled out completely.

Finally, it needs to be noted that reliability was low for some tasks, for example for the ANS task, but also the number line estimation task. This may be due to the small number of trials in these tasks (see Krajcsi et al., 2024). In the ANS task, there were only four trials per ratio and size control condition and there were only three trials for the number line estimation task covering the range from 0 to 10 and six trials for the range from 0 to 100. Initially, we decided to use these small numbers of trials to keep testing time appropriate for the assessed age group. Indeed, low reliability may have constrained the potential to identify significant contributions of the respective tasks with mathematical skills (Krajcsi et al., 2024). At the same time, however, it is important to note that we nevertheless observed significant contributions of both ANS accuracy and number line estimation performance to mathematical skills comparable to previous studies (e.g., Chen & Li, 2014; Gimbert et al., 2019; SchnEider et al., 2018; Van Marle et al., 2014). Reliability of the CE composite score also was low. This may reflect that tests on the central executive are typically more difficult for children than tests on, for example, phonological WM (e.g., Oesterlen et al., 2018). This means that correlations between different CE subtests were rather low which may explain the low reliability of the CE composite score. All in all, these aspects must be kept in mind when interpreting the results of this study.

#### Future perspectives

To further investigate differential contributions of ANS, number line estimation, and WM to the development of mathematical skills from preschool to primary school, future studies may consider more age groups in closer succession, especially for primary school grades 1 to 4. Thus, for example, it may be desirable to investigate whether there is a specific point in time in primary school at which number line estimation performance gains specific importance as a predictor for mathematical skills.

Moreover, besides ANS, number line estimation, and WM, contributions of other early mathematical skills such as symbolic magnitude comparison (Brankaer et al., 2014; Toll & Van Luit, 2014; Toll et al., 2015; Xenidou-Dervou et al., 2018) or number order processing (Lyons & Beilock, 2011; Lyons et al., 2014; Morsanyi et al., 2017) to the development of mathematical skills is discussed. Therefore, future studies may well evaluate potential (differential) contributions of these and other early mathematical skills.

As a reason for the heterogeneous findings concerning the role of ANS for the development of mathematical skills, methodological differences in the ANS tasks are discussed (Dietrich et al., 2015). Some studies for example used approximate addition tasks instead of a non-symbolic comparison task to assess ANS accuracy (Barth et al., 2005; Coolen et al., 2022; Gilmore et al., 2010; Iuculano et al., 2008). By using both kinds of tasks in future studies, different aspects of ANS accuracy may be assessed and compared regarding their contributions to mathematical skills.

Finally, this study indicates that neither number line estimation nor CE appear to mediate the contribution of ANS to mathematical skills. In addition to number line estimation and CE, the literature also suggests other mediators for this association. For example, in the study of Scalise and Purpura (2022) mathematical language was a significant mediator in preschool children. Therefore, more studies investigating different mediators simultaneously with the focus on developmental changes within the acquisition of mathematical skills are needed.

## Conclusions

The present study aimed at evaluating differential contributions of ANS, number line estimation, and WM to mathematical skills in preschool and primary school children likely induced by the start of formal (symbolic) mathematical instruction. Findings indicated that ANS, number line estimation, and WM were significant predictors for mathematical skills in preschool and primary school, when controlled for sex and nonverbal intelligence. Additionally, the results revealed differences in the predictive power of number line estimation for mathematical skills between preschool and primary school children. Number line estimation was a significantly stronger predictor for mathematical skills in primary school children compared to preschool children. In contrast, there were no indications of similar differential contributions of ANS and WM. Taken together, the results of the current study seem to suggest that the start of formal (symbolic) mathematical instruction increases the predictive power of number line estimation which basically reflects the mapping of symbolic onto non-symbolic magnitudes and thus an integration of ANS and symbolic numerical representations.

## Data Availability

Data and code are provided via OSF (10.17605/OSF.IO/39THK).
